# Production of Cyclic Anhydride-Modified Starches

**DOI:** 10.3390/polym13091504

**Published:** 2021-05-07

**Authors:** Ryan C. Amos, Julien Mesnager, Michael Kuska, Mario Gauthier

**Affiliations:** 1 Department of Chemistry, Institute for Polymer Research, University of Waterloo, 200 University Avenue West, Waterloo, ON N2L 3G1, Canada; r2amos@uwaterloo.ca; 2 EcoSynthetix Inc., 3365 Mainway, Burlington, ON L7M 1A6, Canada; jmesnager@gmail.com (J.M.); mike.kuska@gmail.com (M.K.)

**Keywords:** starch, alkenylsuccinic anhydride (ASA), esterification, soybean oil, reactive extrusion

## Abstract

Modified starches offer a biodegradable, readily available, and cost-effective alternative to petroleum-based products. The reaction of alkenylsuccinic anhydrides (ASAs), in particular, is an efficient method to produce amphiphilic starches with numerous applications in different areas. While ASAs are typically derived from petroleum sources, maleated soybean oil can also be used in an effort to produce materials from renewable sources. The reaction of gelatinized waxy maize starch with octenylsuccinic anhydride (OSA), dodecenylsuccinic anhydride (DDSA), a maleated fatty acid (TENAX 2010), phthalic anhydride (PA), 1,2,4-benzenetricarboxylic acid anhydride (trimellitic anhydride, TMA), and three maleated soybean oil samples, was investigated under different conditions. To minimize the reaction time and the amount of water required, the outcome of the esterification reaction was compared for starch dispersions in benchtop dispersed reactions, for starch melts in a heated torque rheometer, and for reactive extrusion in a pilot plant scale twin-screw extruder. The extent of reaction was quantified by ^1^H NMR analysis, and changes in molecular weight and diameter were monitored by gel permeation chromatography (GPC) analysis. The outcome of the reactions varied markedly in terms of reaction efficiency (RE), molecular weight distribution, and average hydrodynamic diameter, for the products derived from the different maleated reagents used, as well as for the different reaction protocols.

## 1. Introduction

**Figure 1:**
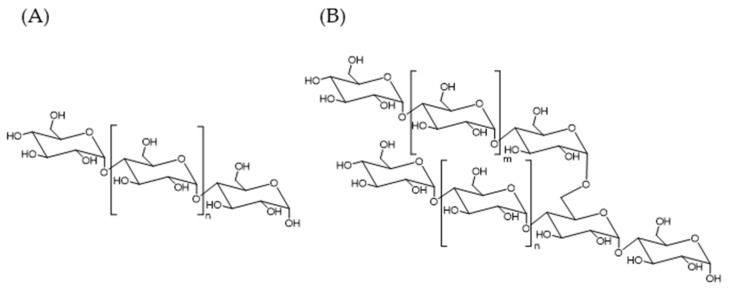
Chemical structure of (**A**) amylose and (**B**) amylopectin.

Starch is a biopolymer that is renewable, readily available, biodegradable and cost-effective [1]. These attributes make it attractive not only for food, but also as a feedstock for industrial applications [2]. Common sources of starch include, but are not limited to corn, wheat and potatoes [3]. Starch is biosynthesized as two different macromolecules, namely amylose and amylopectin [4]. Amylose (Figure 1A) is an essentially linear molecule composed of glucopyranose (GPy) units connected by α-1,4 glycosidic linkages [5,6]. Similarly to amylose, amylopectin (Figure 1B) incorporates GPy units connected by α-1,4 glycosidic linkages [7], but also branching introduced through α-1,6 linkages [8]. Amylose is composed of approximately 200–1200 GPy units, whereas amylopectin can have more than 100,000 GPy units per molecule [3]. The proportions of amylose and amylopectin vary with the plant species [9], while some mutant plant strains are enriched in amylose or amylopectin, such as waxy maize starch containing > 99% amylopectin [10].

Amylose and amylopectin chains in starch granules are deposited as alternating semi-crystalline and amorphous layers [11,12]. The semi-crystalline layers contain the linear amylopectin segments, while the amorphous layers are composed of branched amylopectin segments [10]. The location of the amylose component is not as defined as for amylopectin: in wheat starches, it is concentrated in the amorphous layers, in maize starch it is more evenly distributed across the amorphous and semi-crystalline layers, and in potato starches it is crystallized with the linear amylopectin segments [13].

Native starch has several drawbacks for industrial applications; for example, it is brittle unless it is suitably plasticized [14], but can become soft and weak while plasticized [15]. The mechanical properties of starch can also deteriorate upon exposure to water [16]. One solution to address these issues is to hydrophobically modify starch [2]. Octenylsuccinic anhydride (OSA), an alkenylsuccinic anhydride (ASA), has long been studied as a starch modifier [17]. The introduction of OSA groups onto the hydrophilic starch backbone results in an overall amphiphilic polymer. OSA-modified starch is currently FDA-approved for use in food products at up to 3 wt% OSA contents with respect to starch. These starch-based amphiphilic polymers are used to stabilize oil/water emulsions, for the encapsulation of flavors [18] and coatings, and in gel production [5,6]. Some commercial OSA-modified starch products include, but are not limited to, N-Creamer^TM^, Purity Gum^TM^, CAPSUL^TM^, and Hi-CAP^TM^ [6]. For food grade OSA-modified starch, at least 2.7 wt% of the OSA must be bound to the starch [18], which implies a reaction efficiency (RE) of at least 90%. This may be difficult to achieve in practice, because the granular structure of starch physically inhibits the diffusion of the anhydride inside the granules. Gelatinization is the process of disrupting hydrogen bonds within the granule structure, which results in the release of individual amylose and amylopectin chains [19]. The process is irreversible and requires a plasticizer, such as water or glycerol, and heat [20]. The amounts of heat and plasticizer required for full gelatinization varies with the starch composition, amylose being more crystalline and requiring more energy for disruption of the ordered granule structure [21]. Mechanical treatment of the starch is not required but it accelerates the process. After cooling, the gelatinized (cooked) starch thickens but does not display the same properties as native starch granules [20]. Starch is commonly gelatinized on a large scale in batch processes using blenders and melt mixers, and by continuous techniques such as single or twin-screw extruders [21]. Gelatinized starch simply refers to the absence of granules, which can be lost in more than one way, while thermoplastic starch (TPS) is plasticized starch that is gelatinized through thermomechanical treatment. This treatment implies mechanical work on the starch, which ultimately results in a decreased molecular weight for the starch chains [22]. Gelatinized starch and TPS are hydrophilic, but their hydrophilic character and other physical properties can be tuned with hydrophobic reagents such as OSA, since the hydrophobicity of the products increases directly with their substitution level [6].

OSA and other ASAs are produced by the reaction of maleic anhydride (MA) and unsaturated hydrocarbons, typically containing a terminal alkene [23]. They result from an ene reaction between an electron-poor double bond (MA) and a compound with an allylic hydrogen (an alkene) [24]. Terminal alkenes, such as 1-octene for OSA, are derived primarily from petrochemicals [25]. With depleting oil supplies and increasing prices, it would be advantageous to shift to biosourced materials that are still cost-effective [26]. Vegetable oils and their derivatives are renewable, cost-effective, and biodegradable [24]. Depending on their source, vegetable oil triglycerides (TGs) may contain multiple double bonds and allylic hydrogens per molecule. Soybean oil, one of the most readily available vegetable oils, contains, on average, over four double bonds per TG [27].

This study concerns the reaction of starch with different cyclic anhydrides, namely two commercially available ASAs (OSA and dodecenylsuccinic anhydride, DDSA), TENAX 2010 (a commercially available maleated fatty acid), phthalic anhydride (PA), 1,2,4-benzenetricarboxylic acid anhydride (trimellitic anhydride, TMA) and three maleated soybean oil products developed in our laboratory containing 1.1, 2.0 and 2.3 anhydride rings per TG. These reactions are often completed on granular starch in batch stirred reactions for extended times. With granular starch, the reaction is heterogeneous, and requires a lot of water with respect to the starch and a base. In the current study, gelatinized waxy maize starch was used first in “classical” benchtop batch reactions, following methodologies previously reported for modification with ASAs. To implement the procedures on an industrial scale, the reactions were then transferred to a melt mixer, also referred to as a heated torque rheometer or internal roller mixer, to modify the TPS at high solids (80 wt% starch), with and without NaOH. Finally, starch was modified by a continuous pilot plant scale twin-screw extrusion process using DDSA, TENAX and maleated soybean oil containing, on average, 1.1 anhydride rings per TG. While the removal of solvents and contaminants such as catalysts in starch modification often impairs the economic viability of processes [21], the procedures reported herein use only water as solvent and NaOH, so as to minimize the need for purification. The products obtained were characterized by ^1^H NMR spectroscopy and gel permeation chromatography (GPC). The hydrophobically modified starch products synthesized have numerous potential uses as food thickeners, binders in paper making, or as adhesives [6]. In comparison with starch modified with petroleum-based ASAs, maleated soybean oil-modified starch, in particular, has a higher bio-sourced content.

## 2. Materials and Methods

### 2.1. Materials

Waxy maize starch (waxy pearl 1108) was purchased from Cargill Inc. (Burlington, ON, Canada). OSA and DDSA were purchased from Dixie Chemicals (Pasadena, TX, USA), and TENAX 2010 was acquired from MeadWestvaco Corporation (Richmond, VA, USA). The remaining chemicals were purchased from Sigma Aldrich (Oakville, ON, Canada). All chemicals were used as received. Native waxy maize starch was gelatinized by extrusion in a pilot plant scale twin-screw extruder.

### 2.2. Modification of Dispersed Starch with Cyclic Anhydrides (Benchtop Procedure)

Gelatinized waxy maize starch (60.0 g, 0.370 mol GPy units) was dispersed in 120 mL of distilled water (33 wt%) in a glass beaker and the pH was adjusted to 10 using 20% *w*/*v* NaOH. The mixture was stirred with an overhead mechanical stirrer until the starch solution was homogenous. OSA (1.50 g, 7.13 mmol, 2.5 wt% wrt starch) was dissolved in acetone (approximately 50 wt%) before slow addition (over 10 min) to the stirred reaction. The pH was monitored with a pH meter (ThermoFisher Scientific, Mississauga, ON, Canada) and maintained between 9 and 10 over 60 min by addition of 20% NaOH solution. The reaction was stopped by addition of HCl (1.5 M) to adjust the pH to 6.5–7.0. The crude product was dried by heating under an air stream, while a portion was also purified by precipitation in acetone followed by Soxhlet extraction with acetone for 48 h. The solid products were then dried in an oven at 80 °C at reduced pressure overnight. The crude and purified products were analyzed by ^1^H NMR spectroscopy, and the purified product by GPC. The procedure was repeated for anhydride loadings of 5, 7.5, and 10 wt%. The procedure was also repeated with the other cyclic anhydrides (DDSA, PA, TMA, TENAX 2010 and three maleated soybean oils).

### 2.3. Modification of Starch with Cyclic Anhydrides in a Melt Mixer

Uncooked waxy starch (22.0 g, 0.136 mol GPy units) and distilled water (4.4 mL, 0.244 mmol, 20 wt% wrt starch) were loaded into a melt mixer (Half Size Mixer, C. W. Brabender, 30 mL capacity) fitted to an ATR Plasti-Corder Torque Rheometer (C. W. Brabender) preheated to 90 °C by circulating oil. To measure the internal temperature over the duration of the whole reaction (up to 15 min at 40 rpm), the chamber was fitted with a thermocouple at the bottom. After 4 min, OSA (0.55 g, 2.62 mmol) was added slowly to the mixing chamber. After the reaction, the product was removed from the mixing chamber and ground to a fine powder in a coffee grinder. A portion of the crude product was purified by Soxhlet extraction with acetone for 48 h. The solid products were then dried in an oven at 80 °C at reduced pressure overnight. The crude and purified products were analyzed by ^1^H NMR spectroscopy (300 MHz, DMSO-*d_6_*), and by GPC for the purified product. The procedure was repeated for anhydride loadings of 5, 7.5, and 10 wt%. The procedure was also repeated with the other cyclic anhydrides (DDSA, PA, TMA, TENAX 2010, and three maleated soybean oils). All the reactions were completed either without added base or with 1.1 equiv of NaOH with respect to the anhydride. When NaOH solution was added, the amount of distilled water used in the procedure was decreased so as to maintain the overall water concentration constant.

### 2.4. Modification of Starch with Cyclic Anhydrides in a Pilot Plant Scale Twin Screw Extruder

The modification of waxy maize starch with DDSA, TENAX 2010 or maleated soybean oil containing, on average, 1.1 MA units per TG (1.1 MA/TG) was accomplished by reactive extrusion, similarly to a reported procedure [28,29]. Reactive extrusion was also accomplished while adding 20 wt% NaOH *w*/*w* (1.1 equiv wrt anhydride) to the starch melt during extrusion. The overall amount of water added was adjusted to remain at a consistent level for all the products. Extrusion-modified starch samples were ground into a fine powder with a coffee grinder, a portion was purified by Soxhlet extraction with acetone for 48 h, and the samples were dried at reduced pressure in an 80 °C oven overnight. The crude and purified products were analyzed by ^1^H NMR spectroscopy (300 MHz, DMSO-*d_6_*), and the purified product by GPC.

### 2.5. H NMR Analysis

^1^H nuclear magnetic resonance (NMR) spectroscopy was performed on a Bruker 300 MHz instrument (Milton, ON, Canada). The concentration of all the samples was 15–30 mg/mL in DMSO-*d_6_* with 7 drops of trifluoroacetic acid (TFA), or deuterium oxide for PA- and TMA-modified samples, and 64 scans were averaged. The reported chemical shifts are relative to the residual solvent protons at 2.50 ppm for DMSO-*d_6_* and 4.79 ppm for deuterium oxide.

### 2.6. Gel Permeation Chromatography Analysis

Analytical gel permeation chromatography (GPC) measurements for modified starch samples were performed on a Malvern GPCmax instrument with a TDA 305 triple detector array (Westborough, MA, USA) and one 300 mm × 8.0 mm I.D. PolyAnalytik SuperesTM column (London, ON, Canada) with a theoretical linear PS molar mass range of up to 200 MDa. A flow rate of 0.6 mL/min was used with 0.05 M LiBr in DMSO as the mobile phase at 50 °C. The raw data were collected and analyzed with the OmniSec software package version 5.00. Samples were prepared at a concentration of 2 mg/mL in 0.05 M LiBr in DMSO and filtered through 0.2 μm nylon filters. To obtain absolute molecular weight (MW) values, a pullulan standard with a peak molecular weight M_p_ = 334,000 Da and M_w_/M_n_ = 1.10 (PolyAnalytik) was used to calibrate the instrument. The $\left(\frac{dn}{dc}\right)$ and intrinsic viscosity [η] values supplied for this standard in DMSO were 0.066 mL/g and 0.65 dL/g, respectively.

## 3. Results and Discussion

**Figure 2:**
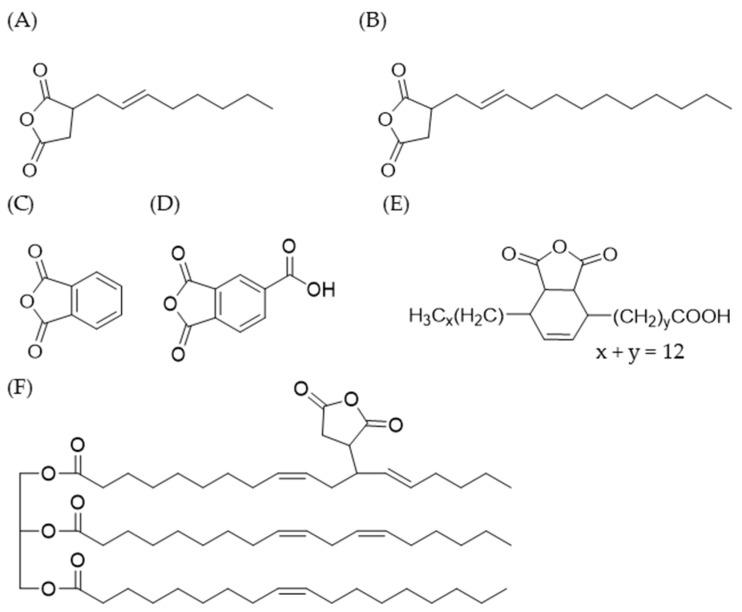
Chemical structure of (**A**) OSA, (**B**) DDSA, (**C**) phthalic anhydride, (**D**) 1,2,4-benzenetricarboxylic acid anhydride, (**E**) TENAX 2010, and (**F**) maleated soybean oil with one equivalent of maleic anhydride (many isomers possible).

The reaction of starch with cyclic anhydrides in the presence of water has been widely investigated, starting with Caldwell and Wurzburg [30] in 1953 who used different alkenylsuccinic anhydrides and sodium carbonate as a base. The reaction mixture was a “slurry”, in that granular starch was simply suspended in water [31]. After 14 h, the reaction was neutralized with hydrochloric acid (HCl) to reach a final pH of 7.0, and the solid product was collected by gravity filtration and washed with either water or ethanol. While this method has been modified to some extent, it is still widely used to produce hydrophobic starch nowadays. One drawback of that procedure is that since granular starch is used, the GPy units on the surface are free to react while GPy units inside the granules are inaccessible. The hydrophobic anhydride must diffuse through the hydrophilic starch granule to react [32]. The longer the chain length, the more hydrophobic the anhydride, which makes this process more difficult. One solution to this problem is to use gelatinized starch, so that the amylose and amylopectin molecules are free in solution and able to react with the anhydride [33]. In the current study, gelatinized waxy maize starch was modified with different cyclic anhydrides. The esterifying agents investigated were OSA, DDSA, TENAX 2010, PA, TMA, and three maleated soybean oil products developed previously in our laboratory (Figure 2). The reaction time for starch modification was set to one hour in the dispersed state. To further optimize esterification, the state of the system was changed from a dispersed phase to a melt phase. Industrial starch esterification is typically achieved in a continuous twin-screw extruder [21] as this method does not require that the starch be dispersed in a solvent, only that it be plasticized [34]. Correspondingly, some reactions were first carried out in a melt mixer as batch reactions under shear, to mimic TPS preparation conditions achieved in a twin-screw extruder, and over less than 15 min. The native waxy maize starch granules were gelatinized in situ under high shear before addition of the anhydride [35], and the reactions were completed with and without base. The starch was also modified in a pilot plant-scale continuous twin-screw extrusion process with DDSA, TENAX and maleated soybean oil containing 1.1 MA/TG. The extent of reaction was determined by ^1^H NMR analysis, while GPC measurements were used to determine whether the starch products suffered chain scission during the modification procedure.

### 3.1. Reaction of Starch with OSA and DDSA in Dispersions and in the Melt Mixer

**Scheme 1:**
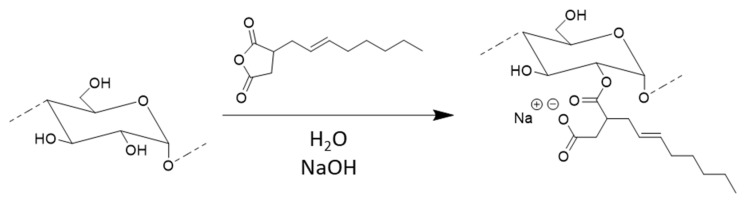
Reaction of starch with OSA. The ester is drawn in the C2 position, but esterification is possible at C2, C3 or C6.

To study the reaction of OSA with starch (Scheme 1), gelatinized waxy maize starch was first dispersed in water at 33 wt% solids content at room temperature, and the pH was adjusted to 10.0 through drop-wise addition of 20 wt% NaOH. When a homogenous dispersion was obtained, OSA diluted with acetone (to lower the viscosity and facilitate its controlled addition, and avoid a high local concentration of anhydride) was added drop-wise to the reaction mixture. The pH of the reaction was maintained between 9 and 10 through the addition of 20 wt% NaOH during this process. The reaction was stopped after 1 h by adjusting the pH to 6.5–7.0 with 1.5 M HCl. After neutralization, a small sample was removed and dried without purification. The purification of another portion of the crude product was achieved by precipitation in acetone, followed by Soxhlet extraction with acetone for two days with occasional stirring to break up clumps, and then drying in a vacuum oven for 16 h at 80 °C. To determine the RE, ^1^H NMR analysis was completed on the unpurified and purified products for each reaction. The spectra obtained for OSA-modified starch (Figure S1) were consistent with those found in previous reports [36]. The peaks between 3 and 4 ppm correspond to protons on the starch backbone, while the peak at 5.1 ppm, for the anomeric proton of starch, can serve as a reference to compare with the integrated signals for the hydrophobic substituents. The methyl signal at 0.8 ppm can serve to quantify the hydrophobic alkyl chains in the mixtures. The peak at 1.3 ppm is for methylene protons in the alkyl group, while the resonance at 1.9 ppm is for aliphatic methylene protons α to carbon-carbon double bonds. Finally, the signals at 5.35 and 5.5 ppm are for alkene protons. These peaks can potentially interfere with the starch anomeric proton signal (5.1 ppm), although at low anhydride loadings this should not be significant. The RE was determined by dividing the integral ratio for the peaks at 0.8 ppm and 5.1 ppm for the purified product through the same integral ratio for the unpurified product, multiplied by 100%. The procedure was completed for 2.5, 5, 7.5, and 10 wt% OSA loadings with respect to starch.

**Figure 3:**
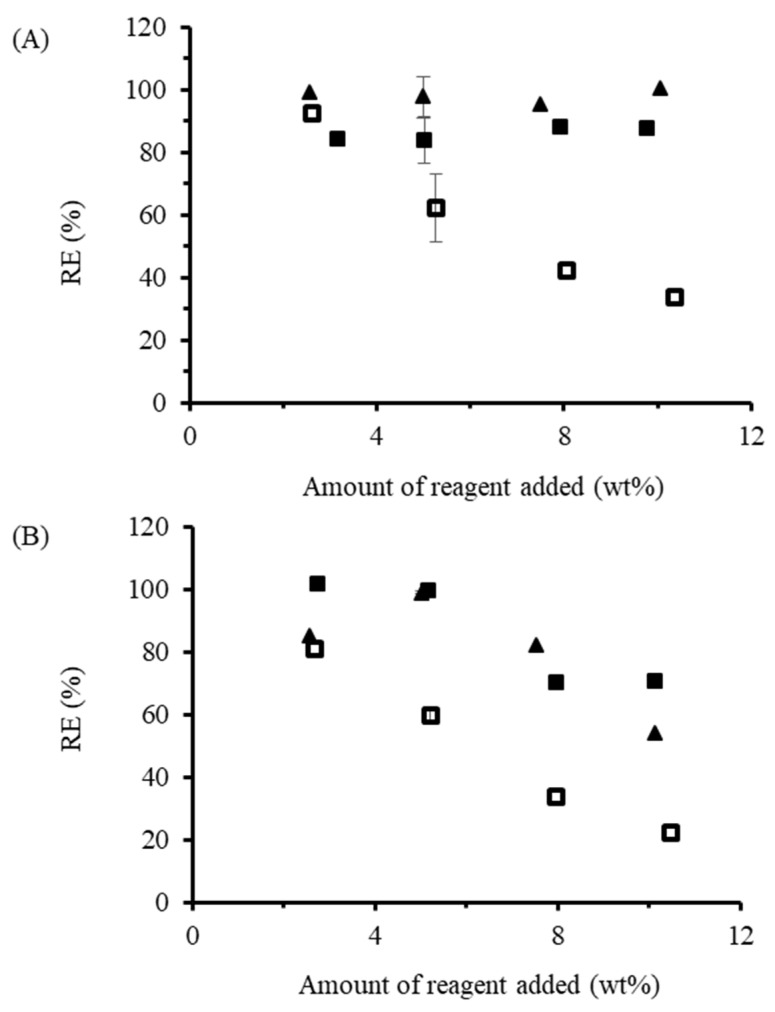
Modification of starch with (**A**) OSA and (**B**) DDSA. Conversion at different weight loadings for (▲) gelatinized starch dispersions, (□) melt mixer reactions without base, and (■) melt mixer reactions with base.

The RE for OSA-modified starch (Figure 3A) prepared in the dispersed phase was 99.1% at 2.5 wt% loading. Increasing the amount of OSA used did not change the RE significantly, which remained between 95.6 and 100%. The high RE achieved with OSA in solution under the conditions described herein are consistent with some previous reports, and higher than for others. For example, Miao and coworkers [5] achieved an RE > 95% for gelatinized maize starch, and above 80% for waxy maize starch granules in 30 wt% dispersions. Bai and Shi [37] quoted values of RE > 99% for water-soluble starch samples, and above 80% for waxy maize starch granules in 40 wt% dispersions. Qi-he and coworkers [38] reported RE values of up to 83% for potato starch granules in 35 wt% dispersions, decreasing to 33% at 10 wt% OSA loading. He and coworkers [14] achieved REs of up to 78% using rice starch granules in 30 wt% dispersions, while Song and coworkers [39] reported 68.5% RE for gelatinized waxy maize starch vs. 74.6% for waxy maize starch granules, albeit the starch concentrations used were unspecified.

**Figure 4:**
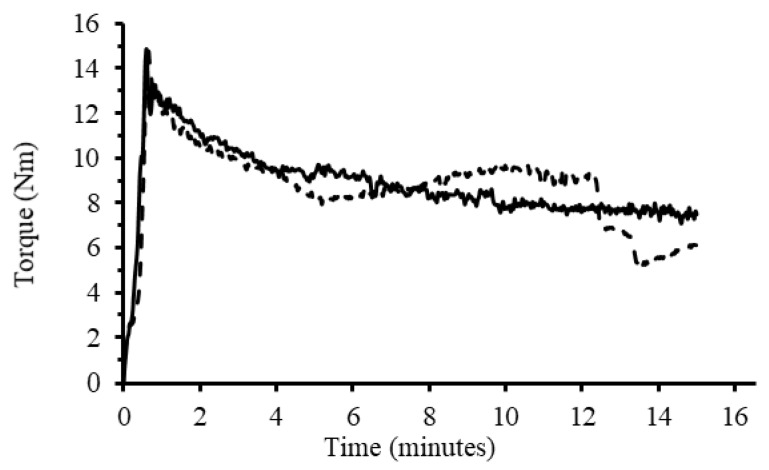
Typical torque variation at 90 °C and 40 rpm for starch with water (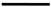) and starch with water and OSA (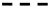).

To complete reactions in the melt phase under homogenous conditions, a melt mixer was initially used. Granular starch was loaded into the melt mixer along with water as plasticizer (20% wrt starch) at 40 rpm. The time and torque recording was started as soon as a torque of 1.0 Nm was obtained (Figure 4). Upon loading of the starch in the melt mixer, there was a sharp rise in torque, followed by a less intense broad peak resulting from water diffusing into the starch granules [35]. The diffusion of water into the granules increased the internal pressure and viscosity within the mixing chamber [7]. After gelatinization, the torque plateaued to a lower value [40]. It is possible for the plasticizer, in this case water, to evaporate at high temperatures, which would result in a slow torque increase [7]. For that reason, a maximum temperature of 90 °C was selected for the reaction with 15 min of mixing to avoid significant water losses. The anhydride was added slowly to prevent pooling of the anhydride in the starch melt, and led to expected small decreases in torque and temperature [7]. As the reaction progressed, the starch melt became more viscous again, which also led to an increase in temperature due to the higher torque. Water condensate was also visible on the mouth of the mixer above the reaction, confirming water losses from the reaction mixture. After the reaction, the modified starch product was removed from the melt mixer, ground into a fine powder, and part of the material was purified by Soxhlet extraction with acetone for two days to remove all unreacted anhydride. Preliminary experiments revealed that dialysis in acetone with three solvent changes was insufficient to achieve similar results. Soxhlet extraction solved this issue by providing continuous solvent exchange. Products were prepared with loadings of 2.5, 5, 7.5, and 10 wt% OSA, similarly to the dispersed phase reactions. The procedure was also repeated using 1.1 equiv of NaOH with respect to OSA in the reaction. The volume of water added initially was reduced by the volume of 20 wt% NaOH solution in that case, so as to maintain 20 wt% water in the reaction.

Increasing the OSA loading led to decreased RE within the 2.5–10 wt% range tested. While the RE at 2.5 wt% OSA loading was 92.6%, close to the 95% RE obtained for the dispersed phase reactions, it did not plateau at higher loadings, as seen in other studies [31]; rather, it dropped to 33.8% at 10% loading. Besides hydrolysis of the anhydride, possible explanations for a drop in RE include the limitations of using a melt mixer to mimic reactive extrusion conditions. It is also possible that the starch was not fully gelatinized, limiting the number of hydroxyl groups available to react. The reaction time was significantly lowered to 11 min, as compared to 1 h for dispersion modification. Longer reaction times may result in higher REs; however, long reaction times cannot be attained with a single pass through an extruder, and thus, these conditions were not pursued. Furthermore, the reaction between the starch hydroxyls and the anhydride depend on the reactivity of the hydroxyl groups. The addition of a base increases the rate of reaction, and literature reports mostly concern reactions completed with a base [21]. The RE with added base was indeed significantly increased, ranging between 80% and 90%, but still lower than for dispersed phase reactions (RE > 95% in all cases).

When the procedures described above were repeated with DDSA, different trends were observed (Figure 3B). For the dispersed phase reactions, an RE of 85.1% was achieved at 2.5 wt% loading, even increasing to 99.0% at 5 wt%. Unfortunately, the reaction mixture began foaming at higher loadings, which suggests DDSA hydrolysis leading to the formation of surface-active sodium dodecenyl succinate. Accordingly, the RE decreased to 82.3% at 7.5 wt% loading, and finally to 54.0% at 10 wt% DDSA. These results are consistent with previous reports on DDSA, such as that of Jeon and coworkers [31], who achieved REs of 80 and 63% at 5 and 10 wt% loadings, respectively, when using waxy maize starch granules with a 6 h reaction time and a starch concentration of 31 wt%. They also found that increasing the DDSA loading beyond 10 wt% did not yield increased substitution levels. Chi and coworkers [16] reported lower RE values of 71.1% and 42.7% at 3 and 10 wt% loadings, respectively, for maize starch granules at 30 wt% concentration.

The RE for DDSA-modified starch in the melt phase without base followed the same decreasing trend as the melt reactions with OSA without base as a function of anhydride loading, except for 5 wt% loading, which was highest. When NaOH was used, 100% RE was achieved for both 2.5 and 5 wt%, but the efficiency dropped to 70% at higher loadings (Figure 3B).

The molecular weight distribution of the modified starch products was determined by GPC analysis using a mobile phase of 0.05 M LiBr in DMSO. The dispersed phase products of OSA and DDSA (Figure S2) displayed no significant differences in their elution profile with respect to unmodified gelatinized waxy maize starch. The unmodified starch had an absolute number-average molecular weight M_n_ = 2.2 × 10^6^ g/mol and an absolute weight-average molecular weight M_w_ = 4.5 × 10^6^ g/mol, corresponding to a polydispersity index Ð ≡ M_w_/M_n_ = 2.0. The weight-average hydrodynamic diameter (D*_h_*) of the molecules was 50 nm. The fact that the elution curves and the molecular weight averages (Table 1) for the unmodified and modified starches only displayed minor variations strongly suggests that no significant degradation or chain scission occurred during the dispersed phase reactions. This is consistent with the report of Miao and coworkers [5], for which a reaction with 3 wt% OSA under similar conditions resulted in no decrease in molecular weight when using gelatinized maize starch; however, there was a significant (57%) decrease in molecular weight when the reaction was completed on waxy maize starch granules.

To generate reference samples for reactions completed in the melt mixer, granular waxy maize starch employed in the melt mixer experiments was processed with water for 15 min under the same conditions as the anhydride reactions. The processed starch prepared at 40 rpm had an absolute M_n_ = 5.6 × 10^6^ g/mol, an absolute M_w_ = 1.9 × 10^7^ g/mol (Ð = 3.4) and D*_h_* = 80 nm, all higher than the materials used in the dispersed phase reactions. The same starch grade was used for the dispersed phase reactions and the melt mixer reactions; however, the starch originated from different lots. The observed differences in absolute molecular weight among the lots are attributed to a combination of variance in year-to-year growth conditions, which have previously been shown to result in more than one order of magnitude difference in M_w_ for maize starch, and the fact that the gelatinized starch was prepared under higher shear conditions in a twin-screw extruder, rather than in a melt mixer [8,11].

Absolute molecular weight analysis was attempted using the light scattering detector on the GPC system for the starch modified on the melt mixer. Unfortunately, the low-angle light scattering (LALS) detector signal was saturated by a large high-molecular weight shoulder (Figure S3), preventing reliable molecular weight measurements. A high molecular weight shoulder was visible in the RI signal for the products independently of the anhydride used, or whether a base was used (Figure S4). While this appears somewhat unlikely under the conditions used, the free carboxylate groups formed in the esterification with the anhydrides could participate in Fischer (also referred to as Fischer–Speier) esterification [41]. The mechanism of this acid-catalyzed reaction involves protonation of the carboxylic acid, followed by intermolecular nucleophilic attack of a starch hydroxyl on the protonated acid, to produce an ester linkage and a water molecule. The acid catalyst is produced by the reaction of starch with the anhydride, at least in the case of reactions not involving a base. In addition to Fischer esterification, there is a potential for dehydration of the carboxylic acid groups, leading to the formation of intermolecular anhydride linkages. Starch has, indeed, been modified with carboxylic acids such as citric acid to cross-link starch via anhydride linkages [42]. These reactions are likewise acid-catalyzed and favored at high temperatures [43]. Considering the very high molecular weight of starch (well over 10^6^ g/mol), the intermolecular formation of ester or anhydride bonds could certainly explain the appearance of the shoulders in the GPC traces of Figure S4.

### 3.2. Reaction of Starch with Phthalic Anhydride (PA) and 1,2,4-Benzenetricarboxylic Acid Anhydride (TMA) in Dispersions and in the Melt Mixer

**Figure 5:**
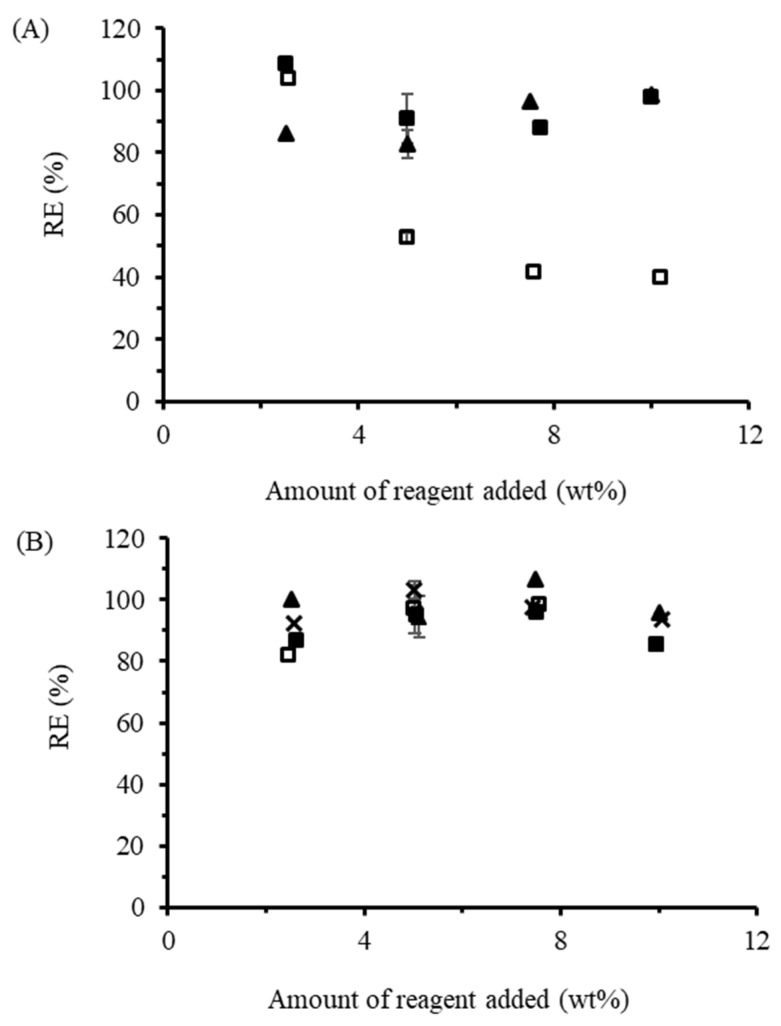
Modification of starch with (**A**) PA and (**B**) TMA. Reaction efficiency at different weight loadings for (▲) dispersed starch, (□) melt mixer reactions without base, (■) melt mixer reactions with 1.1 equiv of NaOH, and (x) melt mixer reactions with 2.2 equiv of NaOH.

While the reaction of starch with OSA or DDSA introduces a hydrophobic alkyl tail, PA and TMA introduce an aromatic ring onto starch. Dispersed phase reactions with PA and TMA were completed similarly to OSA and DDSA, except that PA was not completely soluble in acetone at 50 wt%. For that reason, PA was dissolved in THF (50 wt%) rather than acetone. ^1^H NMR analysis was completed in D_2_O for both PA- and TMA-modified products, because the peak from TFA in DMSO-*d_6_* overlapped with the aromatic signal used for quantification at 7.37 and 7.43 ppm. The RE for the PA derivatives (Figure 5A) followed a trend similar to OSA, with high RE values for the dispersed phase reactions and the base-catalyzed melt phase reactions: a RE of 86.1% was achieved in the dispersed phase at 2.6 wt% loading, increasing to 98.6% at 10 wt% loading. The RE likewise decreased for increasing loadings in the melt phase reactions without base, from an apparent RE > 100% (attributed to integration errors in the ^1^H NMR spectrum) at 2.6 wt% loading, decreasing to 39.9% at 10.1 wt% loading. The corresponding PA reactions in the melt mixer with base also had an RE > 100% for 2.5 wt%, remaining above 85% at the higher loadings. Only one report has been published on the reaction of PA with starch in the presence of water [44], and it concerned reactions conducted in a twin-screw extruder rather than a batch mixer. Interestingly, it was determined that using either 20 or 30 wt% aqueous sodium carbonate as buffer at 30 rpm and 110 °C, at PA loadings above 2.5 wt%, led to hydrolysis of the anhydride. Reactions completed with 0.5 and 1.0 wt% PA under the same conditions were reported as “near quantitative” by the authors.

The RE for TMA in the dispersed phase (Figure 5B) was above 95% at all loadings tested (2.5–10 wt%), similarly to OSA and PA. For reactions in the melt phase without base, RE values of 92.6, 94.5, 98.4, and 85.3%, were achieved at loadings of 2.5, 5.1, 7.5, and 10 wt%, respectively. There was, therefore, no substantial decrease in RE of the type observed for OSA, DDSA, and PA without base. For the base-promoted reactions, due to the presence of a carboxylic acid group in TMA, the procedure was attempted using both 1.1 and 2.2 equiv of base per anhydride. The first equivalent of base was expected to neutralize the free carboxylic acid, while the second equivalent would neutralize the acid formed during esterification of the starch. With 2.2 equiv NaOH, RE > 90% was achieved, while the RE with 1.1 equiv of base was likewise high, within 5% of the reactions without base. Additional base, therefore, did not lead to much improvement in RE for TMA. To the best of our knowledge, this is the first report on the reaction of TMA with starch under aqueous conditions.

The absolute molecular weight averages (Table 2) and D*_h_* for the PA- and TMA-modified products prepared in the dispersed phase, as with OSA and DDSA, were similar to the unmodified starch. The corresponding RI elution profiles were likewise identical to unmodified starch, indicating that no degradation or cross-linking occurred during the reaction.

The molecular weight and D*_h_* of PA-modified starch prepared in the melt mixer without base decreased considerably with respect to unmodified starch (Table 3): the M_n_ and M_w_ of starch modified with 5 wt% PA decreased by more than one order of magnitude, while the D*_h_* decreased almost 4-fold. The effect was even more pronounced at 10 wt% PA loading, in particular for M_w_, weighted more heavily towards the longer chain components of the molecular weight distribution. Interestingly, the variations in molecular weight averages and D*_h_* for the PA derivatives obtained in the melt mixer with a base did not display the same trends. The 5 wt% PA derivative had a large high molecular weight shoulder in the RI elution curve, resulting in larger molecular weight and D*_h_* values. The 10 wt% PA-modified starch, in contrast, displayed a small decrease in molecular weight and D*_h_*, albeit not comparable with the reaction products obtained without base. A possible explanation for this result is a combination of cross-linking (through intermolecular ester or anhydride bond formation) and chain cleavage occurring during the reaction.

The reactions with TMA followed the same trends observed for PA, with the M_n_ and M_w_ of the products without base decreasing by more than one order of magnitude, and D*_h_* decreasing more than 2-fold. The M_n_, M_w_, and D*_h_* of the TMA-modified products prepared without base were, nevertheless, larger than the corresponding PA derivatives. As for the PA reactions, the decrease in molecular weight and D*_h_* of the products was minimized with base addition, the reactions with 2.2 equiv of base having M_n_, M_w_, and D*_h_* values most comparable to the starch substrate.

While previous reports on starch modification with a base showed signs of degradation [14], this was not observed in the current investigation. If acid-catalyzed hydrolysis was the only cause for the decrease in molecular weight and D*_h_*, the TMA derivatives prepared without base should have a lower molecular weight since unreacted TMA contains a free carboxylic acid group. As that was not the case, hydrolysis cannot be the only factor coming into play. Similarly, the decrease in molecular weight and size did not scale linearly with the TMA loading, as the 5 and 10 wt% TMA products had nearly identical characteristics. This suggests that acid-catalyzed chain cleavage and cross-linking both played a role in the trends observed.

### 3.3. Reaction of Starch with Maleated Vegetable Oil in Dispersions and in the Melt Mixer

All the starch derivatives reported so far were synthesized using anhydrides derived from petroleum products [25]. TENAX 2010 is a commercially available maleated fatty acid derived from tall oil by Ingevity (North Charleston, S.C., USA) [45]. Beyond TENAX 2010, reactions were also completed using three different maleated soybean oil products (Table 4) previously synthesized in our lab. While TENAX is produced from C_18_ fatty acids, maleated soybean oil is an entire TG containing over 50 carbons. The hydrophobic domains introduced in starch by reaction with one mole of maleated soybean oil would, therefore, be much larger than for one mole of the ASAs reported above. While the reaction with starch involved anhydride rings, further reactions of the modified starch could focus on the carbon-carbon double bonds; for example, cross-linking with atmospheric oxygen for coating applications [46].

**Figure 6:**
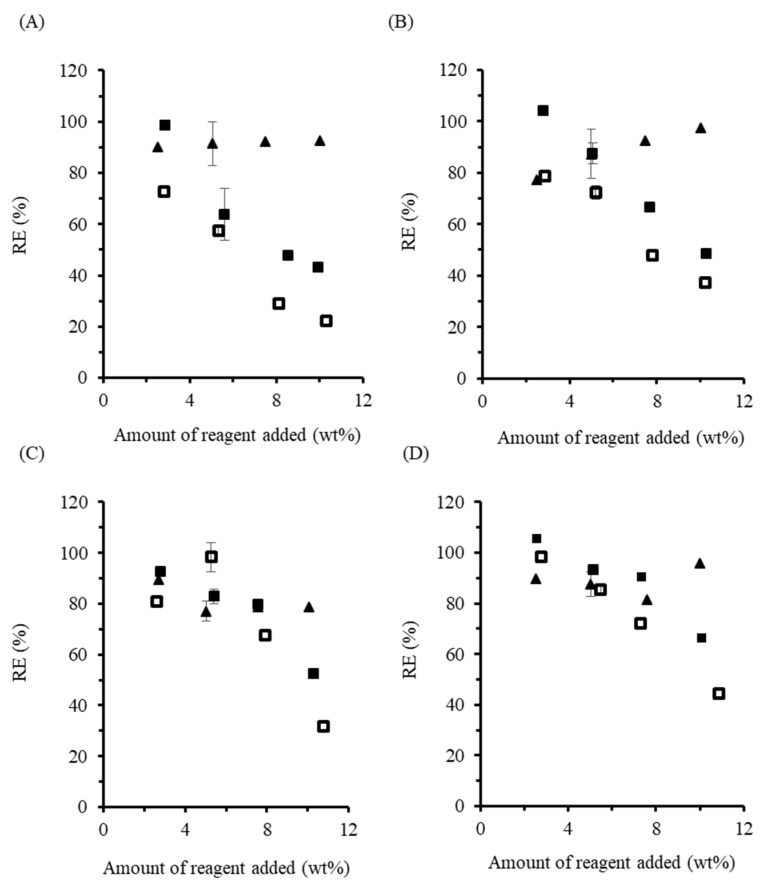
Modification of starch with (**A**) TENAX 2010, (**B**) MSO-1.1, (**C**) MSO-2.2, and (**D**) MSO-2.3. Conversion at different weight loadings for (▲) dispersed starch and melt mixer reactions (□) without and (■) with base.

Reactions between starch and TENAX in the dispersed phase were completed in the same manner as described above, except that the starch was dispersed at 25 wt% instead of 33 wt%. This is because for reactions with 5 wt% TENAX loadings and above, the viscosity of the reaction increased to the extent that the reaction formed a solid mass around the impeller of the mechanical stirrer. This impeded mixing and pH control in the reactions but did not result in a drop in RE, which remained above 90% at all loadings (Figure 6A). Foaming of the type observed with hydrophobic DDSA (containing a C_12_ alkyl tail) was not observed for reactions with TENAX. Foaming was likely suppressed due to the higher RE for the TENAX reactions, leading to a lower succinate salt concentration in the solution acting as a surfactant, in addition to the increased viscosity. The higher viscosity also suggests that reactions with TENAX may be more suitable for melt mixer or extruder operations, designed for these conditions. To the authors’ best knowledge, this is the first report on reactions between TENAX 2010 and starch.

Modifications to the reaction conditions were also required for the vegetable oil-based anhydrides in the melt mixer, since, at 40 rpm, the torque immediately dropped to zero upon addition of the oil, indicating that homogeneous mixing was not achieved. This problem was avoided when the reactions with vegetable oil-based anhydrides were completed at 60 rpm. The reactions in the melt phase, with or without base, followed trends similar to DDSA since increasing the loading of anhydride resulted in a decrease in RE. The highest RE achieved for reactions without base varied from 72.9 to 22.3%, and from 98.6 to 43.0% with base. In contrast to reactions completed in the dispersed phase with gelatinized starch, reactions in the melt mixer had uniform mixing throughout the whole procedure. Similarly to the OSA, DDSA and PA reactions, a base led to higher RE values.

Dispersed phase reactions between starch and the maleated soybean oils were completed as described for TENAX since at weight loadings of 5 wt% and above, the reaction mixture likewise formed a solid mass around the impeller of the stirrer. For dispersed phase reactions with MSO-1.1, an RE of 77.2% was achieved at 2.8 wt%, increasing to 97.3% at the highest loading of 10.0%. The two remaining maleated oils did not show the same RE dependence on oil loading, as, for MSO-2.0, an RE of 89.7% was achieved at 2.7 wt%, decreasing to 78.0% at higher loadings, while for MSO-2.3, the lowest RE of 81.4% was obtained at 7.6 wt% loading and the highest RE was 95.9% 10.0 wt% loading.

**Figure 7:**
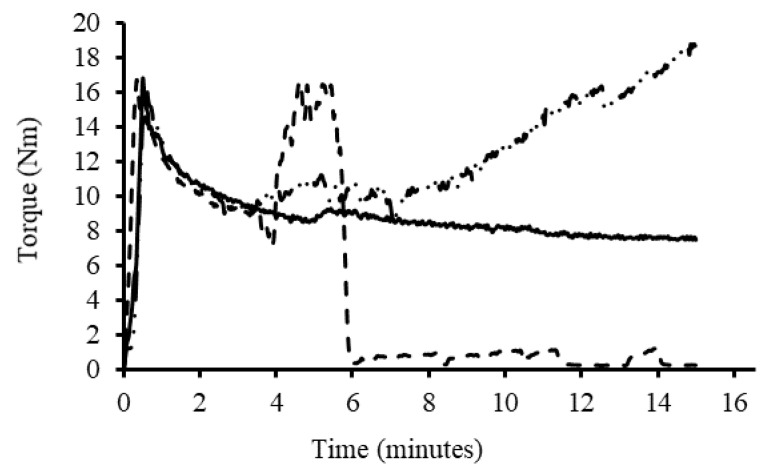
Typical torque curves at 90 °C and 60 rpm for starch with water (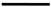), and for starch, water and MSO-2.0 without base (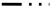) and with base (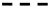).

The melt phase reactions with maleated soybean oil also proceeded differently: while a small decrease in torque was observed upon addition of the other reagents to the melt mixer, maleated soybean oil yielded an increase in torque (Figure 7). Such an increase in torque is characteristic for starch cross-linking [48]. In the absence of base, the torque increased gradually throughout the reaction, reflecting increased shear forces on the starch derivative. With added base, the torque increased more rapidly. Within 2 min from the sharp torque increase, the plasticized starch returned to a powder form and the torque dropped to near zero since melt mixing was no longer achieved. Due to the loss of melt integrity when a base was added, the maleated oil samples had less time to react in a homogenous phase, in contrast to the other anhydrides, which had approximately 11 min to react in the melt phase. The torque increase prior to melt breakdown was proportional to the maleation level of the soybean oil, which is further evidence for cross-linking. In spite of the melt breakdown issue, the RE remained higher in the presence of a base: while for MSO-1.1, the RE decreased from 78.7% at 2.9 wt% to 37.1% at 10.2 wt% loading without base, the RE increased “above 100%” at 2.8 wt% and decreased to 48.7% at 10.3 wt% loading when base was used. Similarly, for MSO-2.0 without base, the RE was highest (95.4%) at 5.3 wt% loading, decreasing to 31.6% at a 10.8 wt% loading. When base was used, the RE varied from 92.5% at 2.8 wt% loading to 52.5% at 10.3 wt% loading. Thus, despite the significantly reduced time spent in the melt phase, a higher RE was achieved in the presence of a base at all but the 5.3 wt% loading level. Finally, for MSO-2.3 without base, an RE of 98.3% was achieved at 2.8 wt% loading, decreasing to 44.5% at 10.9 wt% loading, while, with base, the RE varied from 100%, within error limits, to 66.4% over a similar composition range. Even though increasing the MA content in the maleated soybean oil product resulted in a higher RE over a shorter time period, reactive extrusion requires the starch derivative to remain as a melt throughout the procedure. If this cannot be achieved, the reaction of starch with high MA/TG oils in a twin-screw extruder may be troublesome.

The average molecular weight (Table 5) of the TENAX- and maleated soybean oil-modified starch products in the dispersed phase had more variance than the products previously synthesized in the dispersed phase. The TENAX-modified starch had M_n_, M_w_ and D*_h_* values comparable with unmodified starch, and the elution profiles were essentially identical, without the high molecular weight shoulder or large D*_h_* increase that would be expected in the presence of cross-linking.

The starch processed with water in the melt mixer at 60 rpm had decreased molecular weights and D*_h_* values (Table 6) as compared to starch processed at 40 rpm (Table 1), with M_n_ = 3.8 × 10^6^ g/mol, M_w_ = 1.3 × 10^7^ g/mol, and D*_h_* = 62 nm. The decrease in molecular weight was expected, as it has been shown that increasing the specific mechanical energy exerted on starch results in molecules of decreased size [49,50]. Consequently, the molecular weight of TENAX-modified starch produced in the melt phase without base is about double that of unmodified starch, with only a minor increase in D*_h_* (Table 6). A possible explanation for this increase is that the torque dropped significantly upon addition of TENAX to the starch, which may have resulted in reduced chain scission. The peak elution volume and shape of unmodified starch and the TENAX-modified starch products was essentially identical in terms of RI response, indicating that there was no change in molecular weight distribution. While the TENAX-modified starch prepared with added base had a slightly higher molecular weight than unmodified starch, there was likewise no significant change in the RI peak elution volume, again indicating that the addition of base did not cause much change in molecular weight or D*_h_*.

As stated previously, the TG molecules of maleated soybean oil contained more than one anhydride ring and could, therefore, act as cross-linkers. Increasing the MA/TG ratio (oil maleation level) should increase the likelihood of cross-linking. For the lowest MA/TG ratio (MSO-1.1), the starch modified in the melt phase only displayed a slight increase in molecular weight and D*_h_* as compared to unmodified starch (Table 6). There was no change in the RI peak elution volume, indicating a relatively unaffected molecular weight distribution. As in the previous cases, the addition of base resulted in higher molecular weight and D*_h_* averages. For reactions completed without base, there was no change in the RI peak elution volume.

The molecular weight and D*_h_* for MSO-2.0-modified starch at 5 wt% loading prepared without base in the melt phase were slightly lower than for unmodified starch, but these values were much lower at 10 wt% loading without base, with M_n_ decreasing 4-fold, M_w_ decreasing 6-fold, and D*_h_* decreasing 2-fold. The corresponding RI peak was shifted to noticeably higher elution volumes, consistently with the observed decrease in D*_h_*. It should also be noted that the decreases observed were apparently mainly due to degradation of the longer chain components, as there was a major loss in the high molecular weight portion of the distribution. When base was added to the reaction for MSO-2.0-modified starch at 5 wt% loading prepared with base, there was no significant change in either molecular weight or D*_h_*, and the corresponding RI peak elution volume was similar to unmodified starch. The RI elution curve for the MSO-2.0-modified starch at 10 wt% loading prepared with base had a noticeable high molecular weight shoulder, which is likely responsible for the increased M_w_ value.

The MSO-2.3-modified products without base in the melt phase suffered substantial reductions in molecular weight, with M_n_ and M_w_ both decreasing by over one order of magnitude and D*_h_* decreasing 4-fold. The RI peak elution volume increase was consistent with the D*_h_* reduction also observed. As with the MSO-2.0 starch product at 10 wt% loading, the high molecular weight population was strongly affected. Similarly to the MSO-2.0 reactions, the addition of a base to the MSO-2.3 reactions compensated for the molecular weight and D*_h_* reductions, leading to molecular weight and D*_h_* values comparable to unreacted starch.

For the MSO-2.0- and MSO-2.3-modified starch products, the decreases in molecular weight or D*_h_* correlated with the substitution level. Our findings that reactions carried out at high torque led to lower molecular weight products but no increase in substitution level are consistent with previous reports on cross-linked starch prepared under high shear conditions. Song and coworkers [49] indeed determined that upon adding a cross-linker to starch in a twin-screw extruder, lower molecular weight starch products were obtained as compared with starch processed under identical conditions but without cross-linker. Upon addition of the cross-linker, increases in torque and temperature were observed as the reaction between GPy units on different starch chains yielded a network. When subjected to a high torque and temperature, the starch chains were more easily fragmented, resulting in smaller starch molecules. Liu and coworkers [50] also investigated the fate of starch molecules travelling through a twin-screw extruder in the absence of cross-linker, by removing samples at different points along the extruder barrel and measuring their molecular weight. They found that the molecular weight decreased as the starch moved down the barrel. The decrease in molecular weight and size was not instantaneous; rather, time was required for the high molecular weight chains to fragment. Furthermore, chain fragmentation was not evenly distributed across the sample, as longer chains were much more susceptible to degradation. They concluded that fragmentation due to high shear likely occurs near the center of the starch molecules. The resulting products have an intermediate size and, most importantly, the process does not involve random fragmentation, as this would result in a complete shift of the molecular weight distribution to a lower range. The same type of shear-induced degradation was observed for MSO-2.0 and MSO-2.3 melt phase reactions without base. Anhydrides on the same TG should react slowly with different starch chains to form a crossed-linked network under these conditions, as compared with base-promoted reactions. The slow reaction leads to a gradual increase in torque after the addition of the maleated oil, which also promotes starch fragmentation. For reactions with a base, the anhydride reacts more quickly with the starch, as reflected in a sharp torque increase. It is also possible that the reaction produced a rapid increase in temperature, effectively driving off water from the starch melt. Due to the loss of melt integrity early in the reaction, the products obtained with a base did not have enough time to undergo significant fragmentation, similarly to the products removed early in the extruder barrel by Liu and coworkers. While the reactions without base and with base ultimately reached similar maximum torque values, the slow reaction of the anhydride without base would have allowed additional fragmentation to take place, resulting in products with a much lower molecular weight and D*_h_*. While MSO-1.1 also had a functionality greater than one, it likely did not form enough intermolecular cross-links to produce significant torque increases and fragmentation, regardless of whether a base was used.

### 3.4. Starch Modification by Reactive Extrusion

Both single and twin-screw extruders can be used to produce modified starch, in a continuous process, at an industrial scale [22]. They can mix starch and other viscous materials in a homogenous and controlled manner [51]. Water and other starch plasticizers can be used at low weight loadings under these conditions, which results in a higher starch concentration. The short residence time, low water content and high temperature (in some cases, well above the boiling point of water) used in an extruder, along with high shear mixing, have been shown to yield over 10-fold rate enhancements for esterification reactions as compared with dispersed phase reactions [21]. High temperature and shear enable starch gelatinization early in the extruder barrel. Reactants should be added at a point after full gelatinization is achieved so as to increase the number of hydroxyl groups available, maximize the RE, and yield products with a more homogenous composition [21].

**Figure 8:**
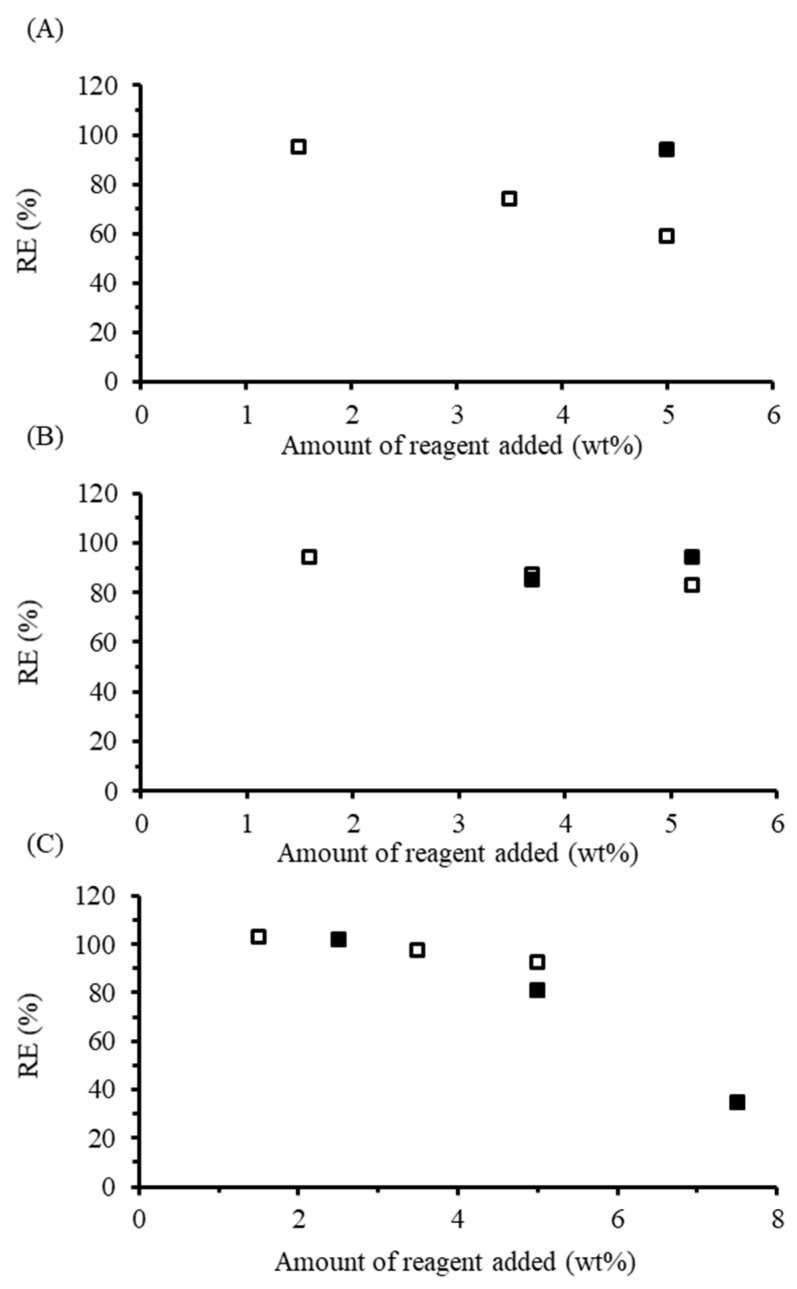
Modification of starch in a pilot plant twin-screw extruder (□) without base and (■) with 1.1 equiv of NaOH and (**A**) DDSA, (**B**) TENAX 2010, and (**C**) MSO-1.1.

When DDSA was used to modify starch in a twin-screw extruder (Figure 8), a trend similar to the reactions in the melt mixer was observed. Without base, an RE of 94.8% was obtained at 1.5 wt% loading, decreasing to 59.4% at 5 wt% loading. When NaOH was added, the RE increased to 93.8% at 5 wt% DDSA. Reactive twin-screw extrusion of regular maize starch with DDSA has been reported by Tian and coworkers [51]. The highest RE achieved in that investigation was 78% using 3 wt% DDSA, 110 rpm, 120 °C, 30 wt% water and 0.5% NaOH. The conditions used in the present investigation, therefore, led to a significant improvement in RE for that system.

For the reactions of starch with TENAX in the twin-screw extruder, RE values higher than for DDSA were obtained. Without base, an RE of 93.8% was achieved at 1.6 wt% loading, decreasing to 83.1% at 5.2 wt% loading. The addition of a base to the reaction did not improve on the 86% RE at 3.7 wt% loading, but increased it to 94.4% at 5.2 wt% loading.

For reactions between starch and MSO-1.1, an apparent RE > 100% was achieved at the lowest weight loading (1.6 wt%), decreasing to 92.2% at a 5 wt% loading. Interestingly, when base was added, a drop in RE was observed, in contrast to the melt phase reactions and the previously discussed extrusion reactions: the RE decreased to 81.3% at 5 wt%, and to 34.9% at a loading of 7.5 wt%. A possible explanation for the drop in RE observed is that the addition of base made the starch melt more hydrophilic, such that the hydrophobic anhydride did not have sufficient time to fully react in the extruder. The conversion of maleated vegetable oil achieved in the twin-screw extruder without base was higher than in the only previous report on that topic. Narayan and coworkers [52], indeed, patented a process involving the modification of starch with maleated corn oil in a twin-screw extruder. The highest RE reported in the patent was 82%, at 4.5 wt% maleated corn oil loading, using 2,5-bis(*tert*-butylperoxy)-2,5-dimethylhexane (Luperox 101) as catalyst and glycerol as plasticizer, although some water was also present in the starch. A higher conversion was achieved herein without catalyst, and using only water as plasticizer.

The molecular weight and D*_h_* of DDSA-modified starch reactive extrusion products were essentially independent of the DDSA loading (Table 7). The reactive extrusion of starch with linear anhydrides was previously reported to result in degradation of the starch [22], but that was not seen here. The addition of base did not cause a decrease in molecular weight or D*_h_*; rather, slightly higher molecular weight and D*_h_* values were observed. The use of base is often cited to cause discoloration in the reactive extrusion of starch, which is attributed to degradation, but no decrease in molecular weight has been reported [21]. The RI peak elution volume for the product prepared with 5 wt% DDSA slightly decreased, which is consistent with higher molecular weight and D*_h_* values.

The TENAX-modified starch prepared in the extruder, both without and with added base, displayed no significant change in molecular weight or D*_h_* at the different substitution levels. For starch modified with MSO-1.1 without base, there was no change in molecular weight or D*_h_*. Only the 5 wt% MSO-1.1 product obtained with a base had lower molecular weight and D*_h_* values than the corresponding product obtained without base, as well as the other MSO-1.1 products. It is also worth pointing out that since the 5 wt% MSO-1.1 starch without base had a higher RE, the difference in molecular weight and D*_h_* does not seem to be directly related to the substitution level.

In the reactions carried out in the melt mixer, the decrease in molecular weight and D*_h_* observed for MSO-2.0 and MSO-2.3 without base is attributed to increased shear forces imposed on the product. The 7.5 wt% MSO-1.1-modified product had a relatively low (34.9%) RE but noticeably higher molecular weight and D*_h_* than the other MSO-1.1-modified products. A possible explanation for the higher molecular weight and D*_h_* of the 7.5 wt% MSO-1.1 product is that since more NaOH was added to the system due to the higher anhydride loading, the addition of NaOH decreased the torque. The addition of NaOH to starch in the melt mixer resulted in a larger drop in torque as compared to deionized water, and it has indeed been shown before that the addition of NaOH lowers the starch viscosity more than pure water [53]. The drop in torque and reduced shear forces exerted on the starch would lead to decreased starch chain degradation.

## 4. Conclusions

Hydrophobically modified starch derivatives were successfully prepared by different methods (dispersed phase, melt mixer and extrusion), by reaction with cyclic anhydrides derived from either petroleum products (OSA, DDSA, PA and TMA) or vegetable oils (TENAX and MSO), through environmentally friendly procedures. The reaction efficiency (RE) was found to depend on the state of the reaction, as well as the structure of the maleated reagent used.

Reactions completed with starch dispersed in water (33 wt% starch) had RE values above 80%, except for DDSA and maleated soybean oil samples. At moderate to high maleated vegetable oil loadings, the reactions were no longer homogeneous. GPC analysis revealed that the molecular weight and hydrodynamic diameter did not increase; therefore, the viscosity increases observed are attributed to the hydrophobic modification.

Reactions completed in a heated melt mixer on starch plasticized with water (80 wt% starch) had decreasing REs for increasing anhydride loadings, except for TMA, which maintained a high RE at all loadings. Reactions completed with a base had higher REs for all the anhydrides tested, indicating that esterification is favored over hydrolysis. Interestingly, the molecular weight and D*_h_* of the modified starch products were greater than for the products prepared without base, in contrast to previous literature reports.

Reactions with maleated soybean oil in the melt mixer led to a significant increase in the measured torque. The base-promoted reactions, in particular, were no longer plasticized, whereas the reactions without base displayed a torque increasing throughout the reaction. GPC analysis revealed that the products of the base-promoted reactions had molecular weight and size characteristics similar to unmodified starch, while the products obtained without base had undergone extensive shear-induced chain scission. The decrease in size observed was attributed to the high shear forces experienced by the starch derivatives, due to the increased torque, rather than to the substitution level.

When comparing the reactive extrusion results with DDSA, TENAX, and MSO-1.1, it should be kept in mind that while the hydrophobicity of the anhydrides increased in the order DDSA < TENAX < MSO-1.1, the RE varied in the same order. Noteworthy is the fact that the MSO-1.1-modified product at 1.6 wt% loading reached 100% RE without a base. The addition of a base increased the RE for DDSA and TENAX while decreasing the RE for MSO-1.1. Reactive extrusion proved to be the most advantageous technique to readily produce hydrophobically modified starch in an environmentally friendly and scalable way. The RE is high enough that the reaction products would not need to be purified before use. A major economic obstacle to the industrial implementation of this modification method is, therefore, removed.

## Figures and Tables

**Table 1:** Absolute molecular weight averages determined by GPC analysis of starch modified in the dispersed phase with OSA and DDSA.

Reagent	Weight Loading (wt%)	M_n_ (g/mol)	M_w_ (g/mol)	Ð	D*_h_* (nm)
N/A	0	2.2 × 10^6^	4.5 × 10^6^	2.0	50
OSA	5	2.2 × 10^6^	5.7× 10^6^	2.7	56
OSA	10	2.5 × 10^6^	5.0 × 10^6^	2.0	54
DDSA	5	2.7 × 10^6^	5.2 × 10^6^	1.9	54
DDSA	10	1.9 × 10^6^	4.0 × 10^6^	2.1	48

**Table 2:** Absolute molecular weight averages determined by GPC analysis of starch modified with PA and TMA in the dispersed phase.

Reagent	Loading (wt%)	M_n_ (g/mol)	M_w_ (g/mol)	Ð	D*_h_* (nm)
N/A	0	2.2 × 10^6^	4.5 × 10^6^	2.0	50
PA	5	1.9 × 10^6^	3.9 × 10^6^	2.1	48
PA	10	1.9 × 10^6^	4.0 × 10^6^	2.2	48
TMA	5	2.2 × 10^6^	4.3 × 10^6^	2.0	48
TMA	10	2.1 × 10^6^	4.2 × 10^6^	2.0	50

**Table 3:** Absolute molecular weight averages determined by GPC for starch modified with PA and TMA in a melt mixer.

Reagent	Loading (wt%)	Base (equiv)	M_n_ (g/mol)	M_w_ (g/mol)	Ð	D*_h_* (nm)
---	0	0	5.6× 10^6^	1.9 × 10^7^	3.4	80
PA	5	0	2.6 × 10^5^	1.5 × 10^6^	5.6	28
PA	10	0	2.3 × 10^5^	9.6 × 10^5^	4.2	24
TMA	5	0	4.7 × 10^5^	1.8 × 10^6^	3.8	31
TMA	10	0	4.4 × 10^5^	1.8 × 10^6^	4.1	31
PA	5	1.1	2.7 × 10^6^	5.8× 10^7^	21.6	103
PA	10	1.1	2.6× 10^6^	8.7 × 10^6^	3.4	55
TMA	5	1.1	1.7× 10^6^	7.1 × 10^6^	4.3	52
TMA	10	1.1	1.7 × 10^6^	1.1 × 10^7^	6.3	57
TMA	5	2.2	4.1× 10^6^	1.2 × 10^7^	3.0	68
TMA	10	2.2	3.2 × 10^6^	1.4× 10^7^	4.4	65

**Table 4:** Maleated soybean oil products previously synthesized in our laboratory used for the modification of starch [47].

Sample	MA/TG	Unreacted MA (wt%)	Unreacted TG (wt%)
MSO-1.1	1.1	1.4	0.89
MSO-2.0	2.0	1.9	0.31
MSO-2.3	2.3	4.6	0.13

**Table 5:** Absolute molecular weight averages of starch modified with TENAX and maleated soybean oil in the dispersed phase.

Reagent	Loading (wt%)	M_n_ (g/mol)	M_w_ (g/mol)	Ð	D*_h_* (nm)
N/A	0	2.2 × 10^6^	4.5 × 10^6^	2.0	50
Tenax	5	2.5 × 10^6^	7.0 × 10^6^	2.8	64
Tenax	10	3.2 × 10^6^	7.8 × 10^6^	2.4	60
MSO-1.1	5	2.0 × 10^6^	4.3 × 10^6^	2.2	48
MSO-1.1	10	2.0 × 10^6^	2.5 × 10^6^	2.2	50
MSO-2.0	5	2.2 × 10^6^	6.9 × 10^6^	3.1	48
MSO-2.0	10	1.9 × 10^6^	7.6 × 10^6^	4.1	56
MSO-2.3	5	1.3 × 10^6^	2.7 × 10^6^	2.0	42
MSO-2.3	10	1.2 × 10^6^	2.3 × 10^6^	2.0	40

**Table 6:** Absolute molecular weight averages and hydrodynamic diameter of starch modified with TENAX and maleated soybean oil in the melt mixer.

Reagent	Loading (wt%)	Base (equiv)	M_n_ (g/mol)	M_w_ (g/mol)	Ð	D*_h_* (nm)
N/A	0	0	3.8 × 10^6^	1.3 × 10^7^	3.3	62
Tenax	5	0	7.9 × 10^6^	2.3 × 10^7^	3.0	90
Tenax	10	0	7.7 × 10^6^	2.0 × 10^7^	2.6	81
Tenax	5	1.1	8.4 × 10^6^	1.9 × 10^7^	2.2	82
Tenax	10	1.1	7.3 × 10^6^	1.5 × 10^7^	2.0	75
MSO-1.1	5	0	6.5 × 10^6^	1.6 × 10^7^	2.5	76
MSO-1.1	10	0	5.6 × 10^6^	1.3 × 10^7^	2.3	70
MSO-1.1	5	1.1	4.9 × 10^6^	2.0 × 10^7^	4.1	81
MSO-1.1	10	1.1	9.0 × 10^6^	2.8 × 10^7^	3.1	99
MSO-2.0	5	0	3.6 × 10^6^	8.8 × 10^6^	2.4	59
MSO-2.0	10	0	8.6 × 10^5^	2.2 × 10^6^	2.6	31
MSO-2.0	5	1.1	3.0× 10^6^	1.6 × 10^7^	5.5	77
MSO-2.0	10	1.1	4.2 × 10^6^	3.1 × 10^7^	7.3	96
MSO-2.3	5	0	1.4 × 10^5^	4.5 × 10^5^	3.2	17
MSO-2.3	10	0	1.1 × 10^5^	3.7 × 10^5^	3.4	15
MSO-2.3	5	1.1	5.2 × 10^6^	1.5 × 10^7^	2.8	63
MSO-2.3	10	1.1	7.8 × 10^6^	2.2 × 10^7^	2.8	73

**Table 7:** Absolute molecular weight averages and D_h_ for starch modified with DDSA, TENAX, and maleated soybean oil by reactive extrusion.

Reagent	Loading (wt%)	Base (equiv)	M_n_ (g/mol)	M_w_ (g/mol)	Ð	D*_h_* (nm)
DDSA	1.5	0	4.3 × 10^6^	7.4 × 10^6^	1.7	64
DDSA	3.5	0	4.7 × 10^6^	8.0 × 10^6^	1.7	66
DDSA	5	0	4.7 × 10^6^	8.2 × 10^6^	1.8	66
DDSA	5	1.1	6.4 × 10^6^	1.5 × 10^7^	2.4	80
Tenax	1.6	0	3.2 × 10^6^	5.1 × 10^6^	1.6	55
Tenax	3.7	0	3.5 × 10^6^	5.8 × 10^7^	1.6	58
Tenax	5.2	0	3.4 × 10^6^	7.2 × 10^7^	2.1	62
Tenax	3.7	1.1	3.7 × 10^6^	7.0 × 10^7^	1.9	61
Tenax	5.2	1.1	3.6 × 10^6^	6.3 × 10^7^	1.7	59
MSO-1.1	1.5	0	2.3 × 10^6^	6.8 × 10^6^	3.0	63
MSO-1.1	3.5	0	1.8 × 10^6^	6.4 × 10^6^	3.5	61
MSO-1.1	5	0	1.8 × 10^6^	6.6 × 10^6^	3.7	59
MSO-1.1	2.5	1.1	3.1 × 10^6^	7.4 × 10^6^	2.4	58
MSO-1.1	5	1.1	1.6 × 10^6^	5.0 × 10^6^	3.1	47
MSO-1.1	7.5	1.1	4.3 × 10^6^	1.2 × 10^7^	2.8	69

## Data Availability

Not applicable.

## Supplementary Materials

The following are available online at https://www.mdpi.com/article/10.3390/polym13091504/s1, Figure S1: ^1^H NMR spectra for OSA-modified starch in DMSO-*d_6_* (top) prior to purification and (bottom) after purification with acetone. Figure S2: GPC elution curves for the baseline-subtracted normalized RI detector response for (a) unmodified gelatinized starch, gelatinized starch modified with (b) 5 wt% OSA, (c) 10 wt% OSA, (d) 5 wt% DDSA, and (e) 10 wt% DDSA in dispersed phase reactions. The position of each curve was shifted on the vertical axis for clarity. Figure S3: GPC elution curves with RI () and LALS () detector responses for starch modified in a melt mixer under identical conditions, leading to LALS detector saturation (top, 5 wt% DDSA without base) and no saturation (bottom, 10 wt% OSA with base). Figure S4: GPC elution curves with baseline-subtracted normalized RI detector response for starch modified in the melt mixer: (a) unmodified starch, and starch modified with (b) 5 wt% OSA, (c) 10 wt% OSA, (d) 5 wt% DDSA, and (e) 10 wt% DDSA without base; starch modified with (f) 5 wt% OSA, (g) 10 wt% OSA, (h) 5 wt% DDSA, and (i) 10 wt% DDSA with base. The position of each curve was shifted on the vertical axis for clarity.
