# Supplementary material for: Production of Cyclic Anhydride-Modified Starches
Source: Polymers (Basel). 2021 May 7;13(9):1504. doi: 10.3390/polym13091504 (PMC8125099; doi:10.3390/polym13091504)
Supplement: Supplementary file 1 [file polymers-13-01504-s001.zip › polymers-1199622-supplementary.pdf]

polymers

Article

Production of Cyclic Anhydride-Modified Starches

Ryan C. Amos [1], Julien Mesnager [2], Michael Kuska [2] and Mario Gauthier [1,*]

[1] Department of Chemistry, Institute for Polymer Research, University of Waterloo, 200 University Avenue West, Waterloo, Ontario N2L 3G1, Canada; r2amos@uwaterloo.ca
[2] EcoSynthetix Inc., 3365 Mainway, Burlington, ON L7M 1A6, Canada
* Correspondence: gauthier@uwaterloo.ca; Tel.: +1-519-888-4567 (ext. 35205)

Supplementary Materials

Citation: Amos, R.C.; Mesnager, J.; Kuska, M.; Gauthier, M. Production of Cyclic Anhydride-Modified Starches. *Polymers* **2021**, *13*, 1504. https://doi.org/10.3390/polym13091504

Academic Editor: Łukasz Klapiszewski

Received: 12 April 2021
Accepted: 4 May 2021
Published: date

Publisher's Note: MDPI stays neutral with regard to jurisdictional claims in published maps and institutional affiliations.

Copyright: © 2021 by the authors. Licensee MDPI, Basel, Switzerland. This article is an open access article distributed under the terms and conditions of the Creative Commons Attribution (CC BY) license (http://creativecommons.org/licenses/by/4.0/).

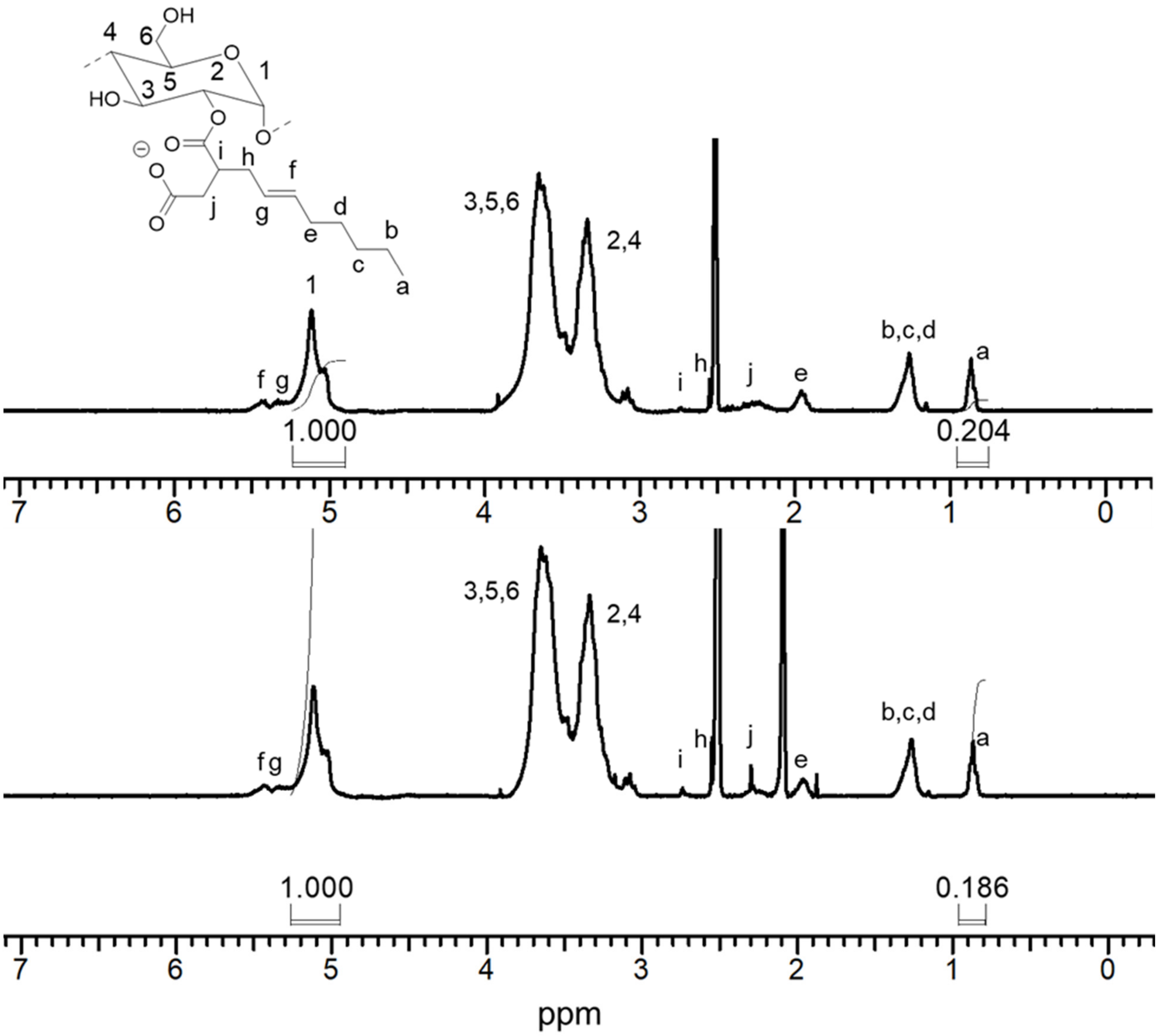

Figure 1. ^{1}H NMR spectra for OSA-modified starch in DMSO-*d*$_6$ (top) prior to purification and (bottom) after purification with acetone.

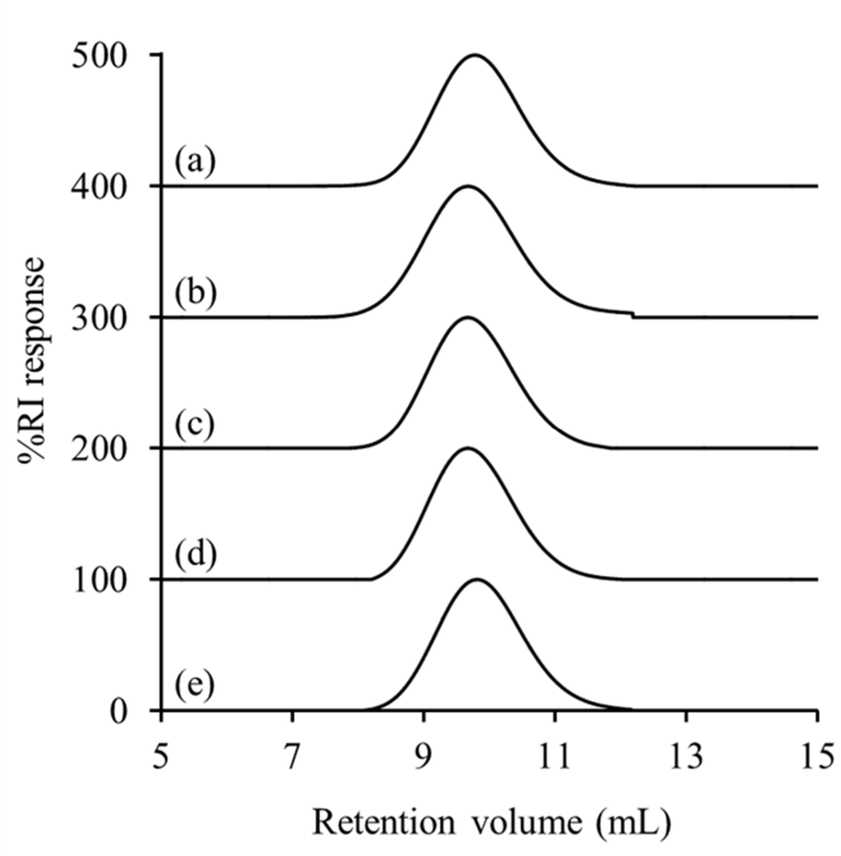

Figure 2. GPC elution curves for the baseline-subtracted normalized RI detector response for (a) unmodified gelatinized starch, gelatinized starch modified with (b) 5 wt% OSA, (c) 10 wt% OSA, (d) 5 wt% DDSA, and (e) 10 wt% DDSA in dispersed phase reactions. The position of each curve was shifted on the vertical axis for clarity.

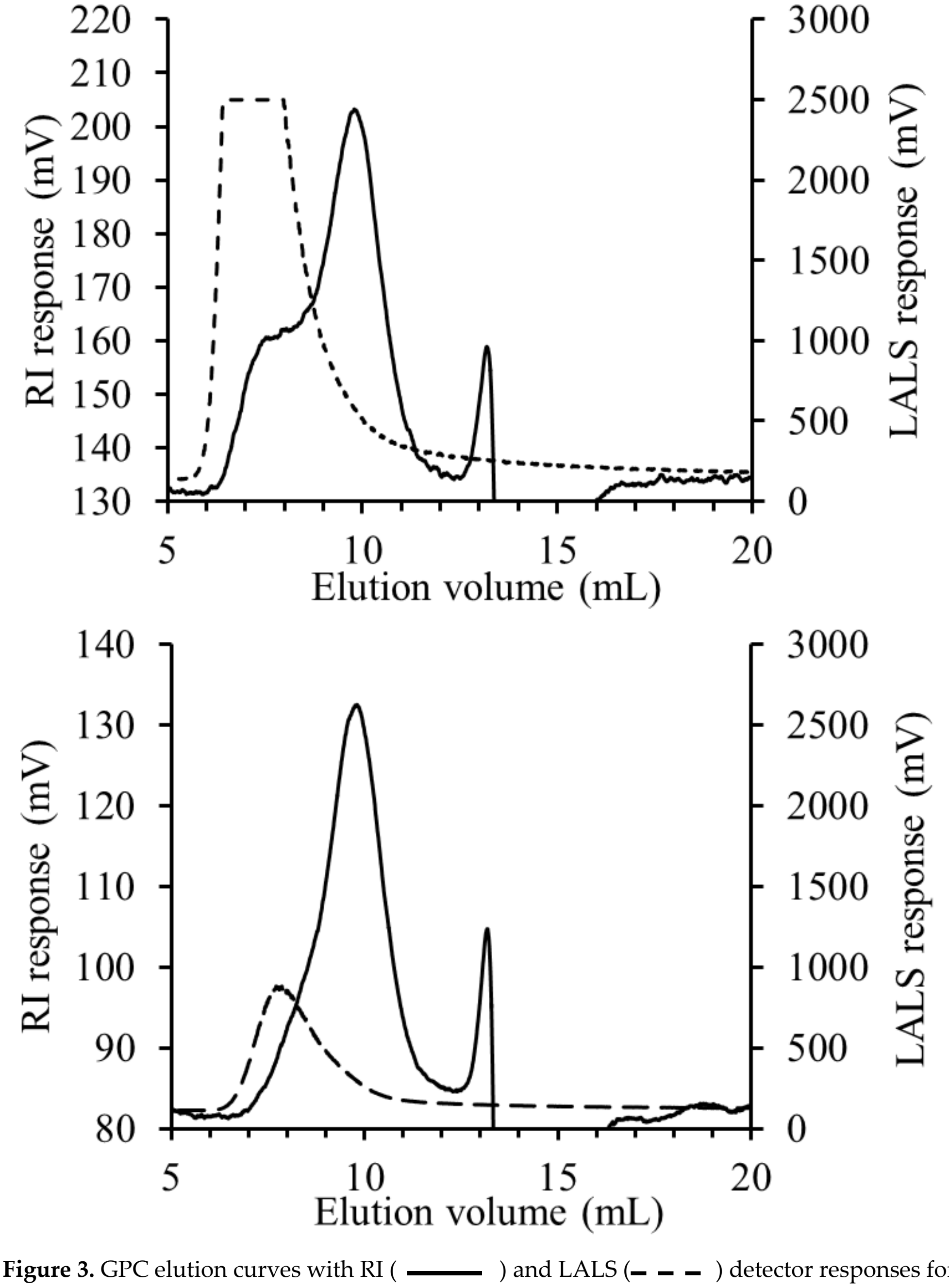

Figure 3. GPC elution curves with RI (———) and LALS (– – –) detector responses for starch modified in a melt mixer under identical conditions, leading to LALS detector saturation (top; 5 wt% DDSA without base) and no saturation (bottom; 10 wt% OSA with base).

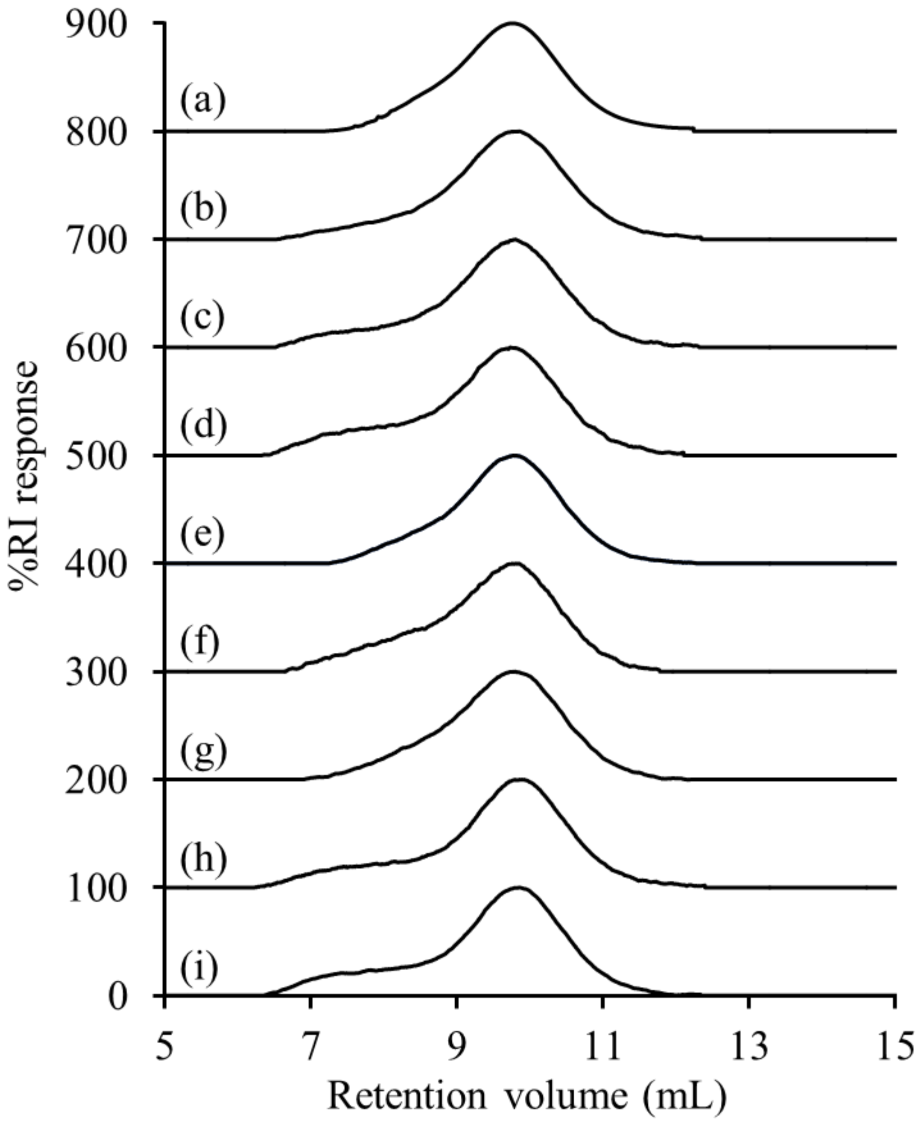

Figure 4. GPC elution curves with baseline-subtracted normalized RI detector response for starch modified in the melt mixer: (a) unmodified starch, and starch modified with (b) 5 wt% OSA, (c) 10 wt% OSA, (d) 5 wt% DDSA, and (e) 10 wt% DDSA without base; starch modified with (f) 5 wt% OSA, (g) 10 wt% OSA, (h) 5 wt% DDSA, and (i) 10 wt% DDSA with base. The position of each curve was shifted on the vertical axis for clarity.
